# DOPO-Functionalized Molybdenum Disulfide and its Impact on the Thermal Properties of Polyethylene and Poly(Lactic Acid) Composites

**DOI:** 10.3390/nano9111637

**Published:** 2019-11-18

**Authors:** Karolina Wenelska, Piotr Homa, Stefan Popovic, Klaudia Maslana, Ewa Mijowska

**Affiliations:** 1 Nanomaterials Physicochemistry Department, Faculty of Chemical Technology and Engineering, West Pomeranian University of Technology, Piastów Ave. 42, 71-065 Szczecin, Poland; phoma@zut.edu.pl (P.H.); kmaslana@zut.edu.pl (K.M.); emijowska@zut.edu.pl (E.M.); 2 Department of Catalysis and Chemical Reaction Engineering, National Institute of Chemistry, Hajdrihova 19, SI-1000 Ljubljana, Slovenia; popovicstefan994@gmail.com

**Keywords:** DOPO, molybdenum disulfide, fire resistance

## Abstract

The fabrication of conventional or biodegradable polymers with improved thermal and fire-resistant properties is an important task for their successful application in various branches of the industry. In this work, few-layered molybdenum disulfide was functionalized with 9,10-dihydro-9-oxa-10-phosphaphenanthrene-10-oxide and introduced into polyethylene and poly(lactic acid) matrixes. The obtained polyethylene composite samples displayed improved thermal stability, significant reduction in CO emissions, improved fire-resistant properties, and over 100% increases in thermal conductivity. Poly(lactic acid) composites displayed less impressive results, but have managed to improve some values, such as CO emissions, peak heat release rate, and total heat release in comparison to pristine polymer.

## 1. Introduction

From household items to high-tech engineering elements, people are bound to use polymers at some point. Since 1939 and the beginning of its industrial use, polyethylene (PE) has become one of the most common plastics [1,2]. It has broad applications in the manufacture of objects like cars, furniture, electrical wiring, insulation, and architectural material [3]. This is due to its outstanding properties, such as a light mass, excellent electric insulation and mechanical durability, good chemical resistance, and ease of processing and molding [4]. However, it has a low melting point and is flammable [4]. Its limiting oxygen index (LOI) value is around 17, which means it can easily burn in open-air conditions [5]. The large-scale use of conventional, petroleum-based polymers has led to an increased amount of polymer waste [6]. Recent developments have been focused on the production of “green” biodegradable polymers [6]. One such material is poly(lactic acid) (PLA), derived from renewable sources, such as corn starch [7]. It serves as a viable alternative to petroleum-based plastics, due to its good biocompatibility [7]. Since its introduction, PLA has found applications primarily in the packaging, medical, and textile sectors [7]. However, its potential for application is limited by its high flammability and melt dripping, which is troubling for electric and electronic industries [8]. Therefore, similar to PE or any other conventional petroleum-based polymers, in order to counteract such flaws, flame retardant (FR) additives are introduced into the polymer matrix of PLA during the production cycle. FRs can be divided into two main groups: reactive (which are chemically bound into the materials) and additive (which are integrated into the materials by mixing only) [9]. Those can act in different ways—they can either interfere with fire’s ability to consume oxygen, form a barrier, or act as a coolant, due to release of products of chemical reactions. Incorporating FR additives into the polymer bulk has been the preferred approach, as it is a relatively easy process that could withstand environmental factors better than the coating of the external surface [10]. This, however, requires very good FR compatibility with the polymer matrix—an ideal FR should not hinder the desirable properties of the polymer and should be easy to process [11]. In addition, the environmental and health profile, as well as costs, should be kept low to reach successful commercial application [12,13]. This means that the development of novel FR systems is a challenging and lengthy process. 

Initially, most common FRs included bromine- or chlorine-based halogenated compounds [14,15]. They came at low cost, but required high loads to meet satisfactory FR performance, which influenced some of the properties of the modified polymers. In addition, it was discovered that the low thermal stability of halogenated FR additives could lead to the formation of toxic and corrosive products during processing or combustion, which were severely harmful to the environment and humans [14,16]. However, it took years for new legislation to be drafted that restricted the use of halogenated FR additives in some applications, and instead promote the development and widespread use of halogen-free FR alternatives [17,18]. This resulted in the recent development of new materials based on phosphorus, nitrogen, and minerals, as well as nanomaterials (i.e. nanoclays, two-dimensional (2D) nanomaterials, and multi-walled carbon nanotubes) that can act as FRs or synergists [19,20,21,22,23,24].

Two-dimensional nanosheets, such as those of molybdenum disulfide (MoS_2_), have found a use in the improvement of thermal, mechanical, conductive, and fire-resistant properties of various polymers [25,26,27,28,29,30]. The high aspect ratio of MoS_2_ nanosheets dispersed in a polymer matrix can act as a physical barrier that retards the diffusion of degradation products, gases, and heat [31]. In addition, molybdenum promotes the formation of a charred layer, which further enhances the flame-retardant effect of the MoS_2_ nanosheets [28]. MoS_2_ nanosheets introduced into polystyrene (PS) matrix resulted in improved thermal stability, fire resistance, and smoke suppression properties, with regard to composites prepared with the use of graphene nanosheets (GNS) [32]. Similarly, a few layered MoS_2_ nanosheets, synthesized by a simple hydrothermal method and introduced into a poly(vinyl alcohol) (PVA) matrix, resulted in improved thermal, mechanical, dielectric, and hydrogen barrier properties, as well as increased char formation in comparison to pristine PVA [29].

Meanwhile, 9,10-dihydro-9-oxa-10-phosphaphenanthrene-10-oxide (DOPO) and its derivatives are known to predominantly act by a gas-phase mechanism through radical quenching mechanics, as a result of PO• radical formation, which might be assisted through condensed-phase action via the incorporation of specific functionalities at the DOPO moiety, or through a synergistic effect with other flame retardants [33,34,35,36]. DOPO functionalized with maleic acid (DOPO-MA) and incorporated into the PLA matrix allowed for the enhancement of mechanical properties and thermal stability, as well as the reduced flammability of composites, compared to DOPO. It was observed that thanks to the modification, DOPO-MA easily reacted with PLA or fibers and formed the charring layer [37]. The addition of 10 wt % of three bis P-C DOPO derivatives to the PLA introduced a gaseous flame inhibition mechanism to the PLA, as well as cross-linked char coating that inhibited the fire with increasing numbers of aromatic groups [38]. This resulted in excellent suppressed dripping efficiency compared to pristine PLA.

In the presented research, little layered 2D MoS_2_ was obtained, and its surface was then functionalized with nickel(III) oxide (Ni_2_O_3_) nanoparticles. Following this, DOPO was grafted onto its surface by linking through Ni_2_O_3_. The resulting MoS_2_/Ni_2_O_3_/DOPO nanohybrids were then introduced into a PE and PLA matrix, in order to obtain composites with improved fire resistance. Here, we provide details of the synthesis, characteristics of obtained nanohybrids, and a full analysis of the thermal and fire properties of the obtained PE and PLA composites.

## 2. Methods

### 2.1. Materials

Bulk MoS_2_ (powder), N-Methyl-2-pyrrolidone (NMP) (anhydrous, 99.5%), and nickel(II) acetate tetrahydrate (98%) were purchased from Merck. PE powder was provided by Merck (Darmstadt, Germany), while PLA powder was purchased from Goodfellow (Huntingdon, England). Hydrogen peroxide (30%), 2-propanol, and anhydrous tetrahydrofuran (THF) were purchased from Chempur (Piekary Slaskie, Poland).DOPO was supplied from TCI America (Tokyo, Japan).

### 2.2. Preparation of Few-Layered MoS_2_

One gram of bulk MoS_2_ powder was dispersed in a mixture of 95 mL of NMP and 5 mL of hydrogen peroxide. Following this, the dispersion was sonicated continuously for 2 h. Following this process, the dispersion was transferred to a round bottomed flask, which was plugged to reflux and continuously stirred for 48 h. Finally, the dispersion was centrifuged four times at 10,000 rpm for 20 minutes and washed with 2-propanol. 

### 2.3. Modification of MoS_2_ with Ni_2_O_3_ Nanoparticles

Obtained few-layered MoS_2_ nanosheets were modified with Ni_2_O_3_ nanoparticles through the hydrothermal method. A total of 250 mg of few-layered MoS_2_ and 250 mg of nickel(II) acetate tetrahydrate were dispersed in 400 mL of 2-propanol and continuously sonicated in an ultrasonic washer for 2 h. Following this, the dispersion was stirred for 48 h, after which 2-propanol was evaporated under room temperature, while the obtained precipitate was dried under a high vacuum at 440 °C for 5 h in a tube furnace. The result of this process was MoS_2_/Ni_2_O_3_ modified nanomaterial.

### 2.4. Functionalization of MoS_2_/Ni_2_O_3_ Nanomaterials with DOPO

The obtained nanomaterial was further functionalized with DOPO. A total of 250 mg of as-prepared MoS_2_/Ni_2_O_3_ nanomaterial was suspended in 100 mL of THF and sonicated for 30 min in an ultrasonic bath. Meanwhile, a 50 mL solution of 3 g of DOPO in THF was also sonicated for the same amount of time. Following this, both solutions were mixed in a round flask, set to reflux, and stirred at 360 rpm in 70 °C for 24 h. After the functionalization process was complete, the mixture was separated by filtration through a 0.2 µm PTFE (Polytetrafluoroethylene) membrane, washed with anhydrous THF and acetone, and dried under a vacuum at 75 °C overnight to remove the remaining solvent. The result of this process was MoS_2_/Ni_2_O_3_/DOPO modified nanomaterial.

### 2.5. Preparation of PE and PLA Nanocomposites

PE and PLA nanocomposites were extruded using a twin-screw extruder (Zamak EHP 2 × 12). For PE, three batches of increasing FR load were prepared: 1 wt %, 3 wt %, and 5 wt %. In the case of PLA, three batches with different FR loads were prepared: 0.5 wt %, 1 wt %, and 2 wt %, respectively. This was in order to verify if it is possible to achieve good FR performance at a low wt % load of FR. For comparison, pristine samples of PE and PLA were also extruded. 

### 2.6. Characterization

The morphology of samples prepared during each stage of the process prior to the extrusion of composites was analyzed using transmission electron microscopy (TEM) (Tecnai F20, FEI, Oregon, USA) with 200 kV accelerating voltage and a scanning electron microscope (SEM; VEGA3 TESCAN, Brno, Czech Republic; high voltage (HV): 30 kV, working distance (WD): 5.25 mm). Thermogravimetric analysis (TGA) was performed using a thermal analyzer (SDT Q600, TA Instruments, New Castle, USA) under airflow of 100 mL·min^−1^, with each sample (ca. 5 mg in alumina crucible) being heated from room temperature to 1000 °C, with a linear heating rate of 10 °C·min^−1^. In addition to this, a Raman analysis was performed in microscope mode (Renishaw, New Mills Wotton-under-Edge, UK) with a 785 nm laser in ambient air. The number of layers for few-layered MoS_2_ was determined with atomic force microscopy (AFM), using a MultiMode 8 microscope from Bruker (Karlsruhe, Germany).

For each composite, TGA was performed, during which the samples were heated from room temperature to 700 °C under airflow, with a heating rate of 10 °C·min^−1^ and air flow 100 mL·min^−1^. Gaseous products from the pyrolysis of composites were analyzed in situ by a mass spectrometer (Pfeiffer Vacuum ThermoStar GmbH, Wetzlar, Germany ) during TGA, conducted under an argon flow of 100 mL·min^−1^ at a heating rate of 10 °C·min^−1^. The peak heat release rate (pHHR), heat release capacity (HRC), and total heat release (THR) of the composites were measured from 2 mg specimens (three samples for each wt % load of FR) through microscale combustion calorimetry (MCC), with use of an FAA Micro Calorimeter manufactured by FTT. The thermal conductivities of the composites, as well as that of pristine PE and PLA, were measured using a laser flash apparatus (XFA 300, Linseis, Selb, Germany). In order to facilitate absorption of the laser on the surface the samples were coated with a thin layer of graphite prior to measurement.

## 3. Results and Discussion

**Figure 1:**
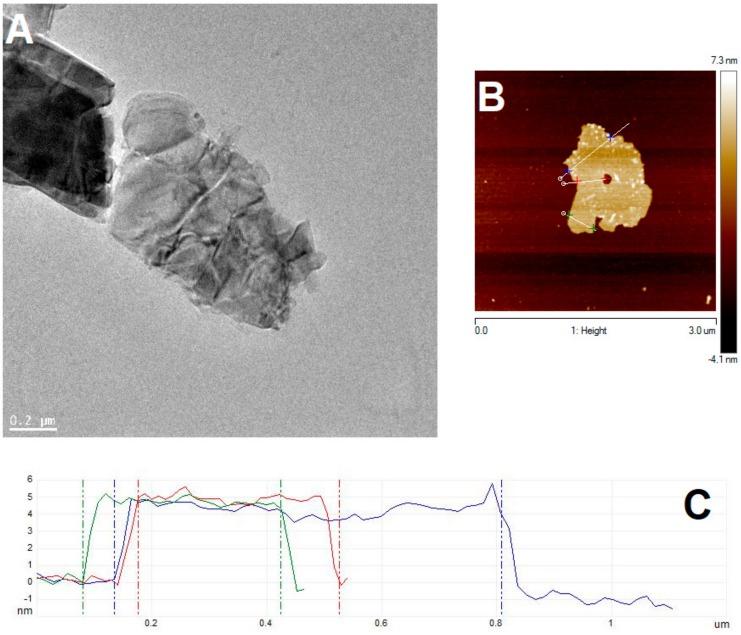
Transmission electron microscopy (TEM) (**A**) and atomic force microscopy (AFM) (**B**) images, along with AFM height profiles (**C**) of few-layered MoS_2._

In order to verify the successful preparation of few-layered MoS_2_, TEM and AFM analyses were performed. As can be seen in Figure 1, few-layered 2D flakes of MoS_2_ were obtained. Height profiles obtained with AFM (Figure 1C) showed an average value of approximately 5 nm, which corresponded to seven layers of MoS_2,_ assuming that the average height of one layer is approximately 0.7 nm [39]. 

**Figure 2:**
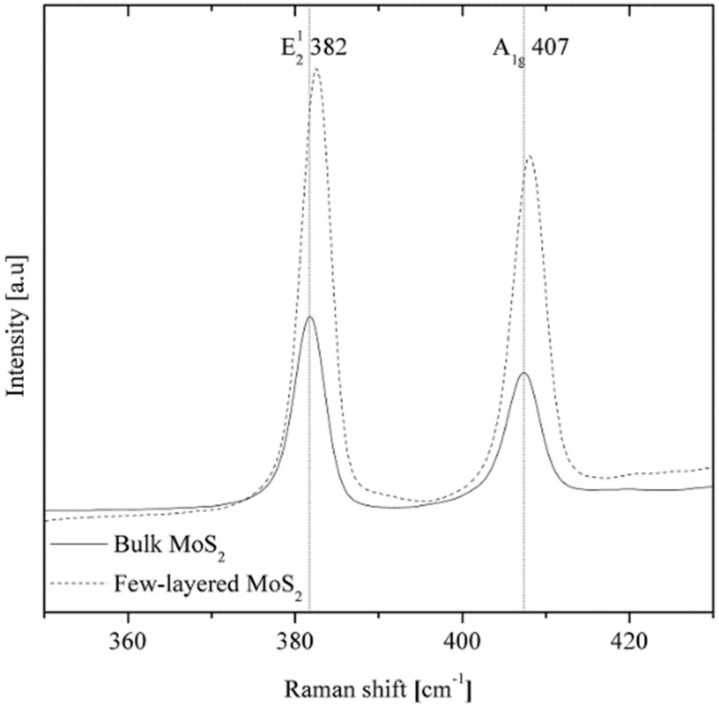
Raman spectroscopy of bulk and few-layered MoS_2_.

Raman spectrometry was utilized for the verification of these results, as presented in Figure 2, and it confirmed that few-layered structures of MoS_2_ were obtained through sonication from bulk MoS_2_. This was visible through a change in the intensity of $E_{2g}^{1}$ (originating from the opposite vibration of two S atoms with respect to the Mo atom) and $A_{1g}$ (associated with the out-of-plane vibration of only S atoms in opposite directions) peaks, located respectively at approximately 382 cm^−1^ and 407 cm^−1^ in the Raman spectra of bulk and few-layered MoS_2_. The frequencies and intensities of these peaks can be used to verify the number of layers on a MoS_2_ flake [40].

**Figure 3:**
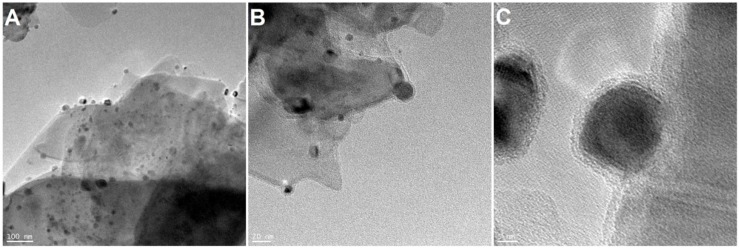
TEM images of MoS_2_/Ni_2_O_3_ nanoparticles (**A**–**C**).

Next, TEM analysis was performed on MoS_2_/Ni_2_O_3_ samples, in order to verify the successful deposition of Ni_2_O_3_ nanoparticles on the surface of MoS_2_ flakes. Ni_2_O_3_ nanoparticle size ranged from 5 to 25 nm on average, as presented in Figure 3. Nanoparticles were distributed on the surface of the MoS_2_ flakes. 

**Figure 4:**
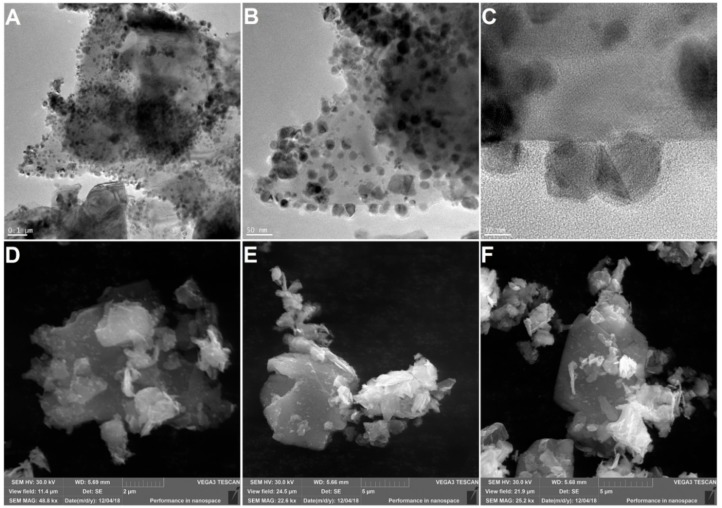
TEM (**A–C**) and SEM (**D**–**F**) images of MoS_2_/Ni_2_O_3_/DOPO nanoparticles.

TEM and SEM analyses of MoS_2_/Ni_2_O_3_/DOPO nanomaterial were also performed, and the collected images are presented in Figure 4. From the images, it appears that DOPO crystals were linked to the MoS_2_ surface through Ni_2_O_3_ nanoparticles, which confirmed the successful functionalization of MoS_2_ flakes. 

### 3.1. Thermal Stability

**Figure 5:**
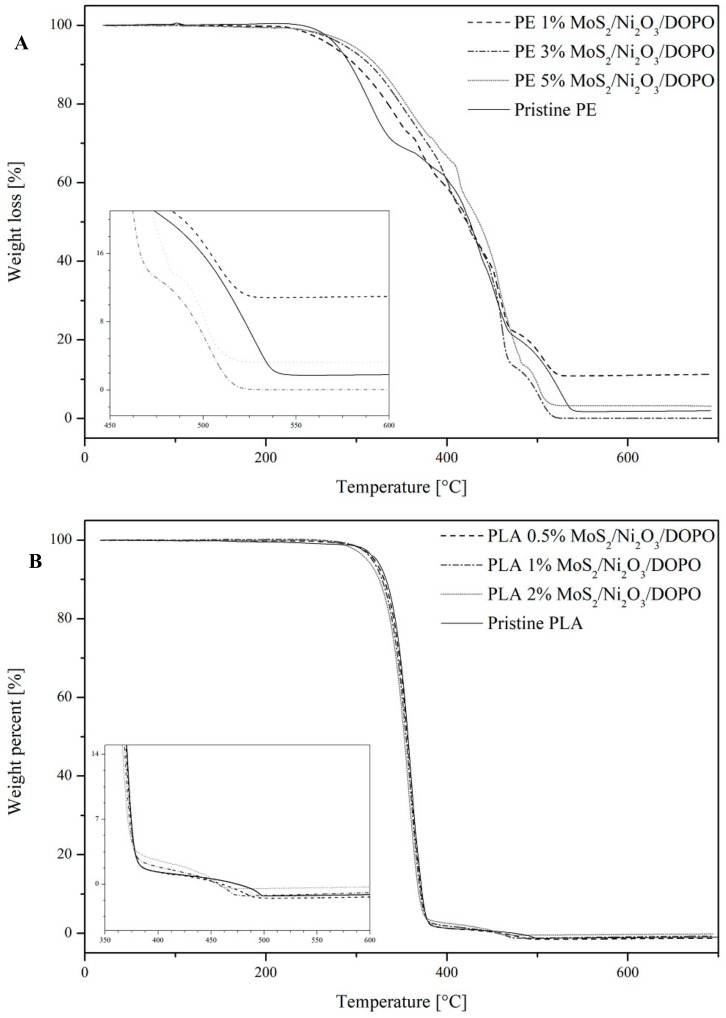
TGA curves of PE (**A**) and PLA (**B**) composites compared to pristine polymers.

The thermal stability of PE and PLA composites containing an addition of MoS_2_/Ni_2_O_3_/DOPO.

(SEM images Figure S1) was analyzed through TGA and compared to pristine samples of polymer. Accumulated data was presented in Figure 5 and Table 1. It can be observed that the temperature (*T*) values for *T_10wt_*_%_, *T_50wt_*_%_, and *T_max_* of the polymer composites changed with the addition of MoS_2_/Ni_2_O_3_/DOPO. In the case of PE composite samples, an increase in the *T_10wt%_* values was observed, which is a sign of improved thermal stability of the composites in comparison to pristine PE. The *T_10wt%_* values rose with the increase in the load of FR. The highest *T_10wt%_* was observed for the sample containing 5 wt % load of FR, and was measured at 30 °C above that of pristine PE. The *T_50wt%_* value increased by 11 °C for this same sample, while the remaining two samples showed this value at 2 °C below that of pristine PE, which was deemed insignificant in comparison. The *T_max_* values decreased for all PE composite samples, which suggests that combustion finished at a lower temperature. This was a direct result of the presence of MoS_2_, which has caused the creation of a char layer on the surface that prevented the underlying material from further combustion. The presence of phosphate groups could also facilitate the catalytic degradation of the PE substrate, significantly contributing to the formation of dense and compact carbonaceous char [41]. In addition to this, 2D nanosheets of MoS_2_ also hindered the diffusion of fuel and gaseous products of decomposition, due to creation of tortuous pathways within the polymer matrix, while PO· introduced a radical quenching mechanism [42,43]. Overall, TGA performance of the PE composites under an air atmosphere was still very close to that observed for pristine PE. 

For PLA samples containing the addition of MoS_2_/Ni_2_O_3_/DOPO with an increase in the load of FR, a stable decrease in *T_max_* values was observed compared to the pristine polymer. Meanwhile, *T_10wt%_* and *T_50wt%_* values remained close to that of pristine PLA, suggesting that the addition of FR did not significantly impact thermal stability. For a sample containing 2 wt % load of FR, the *T_max_* value was recorded at 461 °C, 38 °C below that of pristine PLA. This caused the burning process to finish earlier, with charred residue increasing with an increase in the load of FR. However, a fairly negligible amount of residue suggests that this additive predominantly decomposes to form volatile products, which was also reported in case of polyamide 6 samples containing the addition of bridged DOPO derivatives as FR agents [11,44]. 

**Figure 6:**
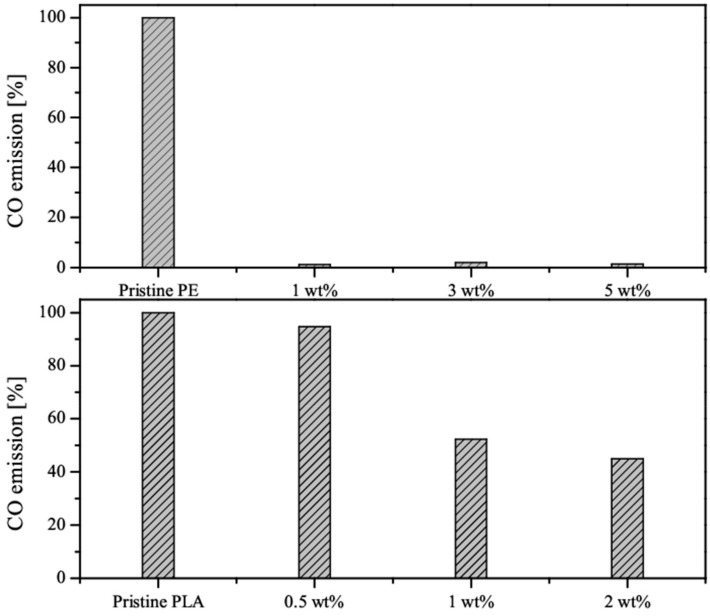
CO emissions of PE and PLA composites compared to pristine polymers.

TGA performed in an atmosphere of argon was used to study CO emissions during pyrolysis of the composites. The results of these tests are graphically presented in Figure 6. In the case of PE composites, a great reduction in CO emissions was observed, ranging up to approximately 98% of value recorded for pristine PE. This result suggests good dispersion of functionalized MoS_2_ sheets inside the polymer matrix of PE, which allowed for the creation of tortuous pathways that hindered diffusion of CO to the surface. This effect was further enhanced by the buildup of a charred layer on the surface of PE, due to the generation and release of molybdenum oxide, as well as the presence of phosphate groups, which introduced radical quenching mechanisms. For PLA composites containing an addition of MoS_2_/Ni_2_O_3_/DOPO as an FR agent, a stable decrease in the emission of CO was observed. The lowest emission of CO (55.1% below that of pristine PLA) was recorded for the sample containing a 2 wt % load of FR. At 1 wt % load, CO emission was reduced by 47.7%. Meanwhile, at 0.5 wt % load of FR, CO emission was only 5.3% below that of pristine PLA. 

The obtained results suggests that a 1 wt % load of FR is the minimal acceptable value for an effective reduction in CO emission during the pyrolysis of PLA. This effect can be mainly attributed to the influence of MoS_2_, which creates tortuous pathways that impact gas diffusion through the structure of polymer, similar to graphene nanosheets [42]. Based on the obtained results, it can be assumed that an FR load of 0.5 wt % seems insufficient for an effective reduction in CO emission from the pyrolysis of PLA.

### 3.2. Flammability Studies

**Figure 7:**
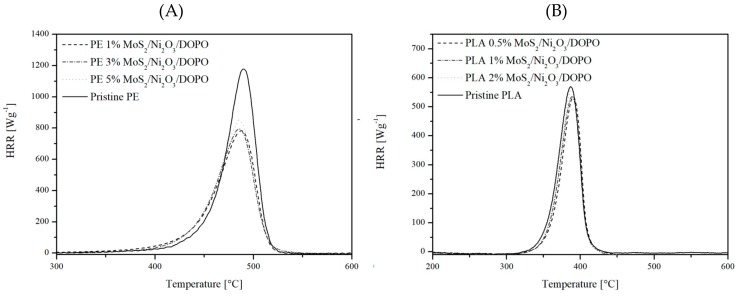
Examples of heat release rate (HRR) curves obtained for PE (**A**) and PLA (**B**) composites compared to pristine polymers.

MCC was employed to evaluate the fire performance of the prepared polymer composites. Heat release curves of the composites are presented in Figure 7, while Table 2 contains data collected from the analysis. In the case of PE, a significant decrease in pHRR (Heat Release Rate pick) values was observed, paired with noticeable drop in THR. 

These results indicate a good fire-resistant performance of the PE composite in comparison to pristine PE (data for individual components Table S2, Figure S2), due to the development of a stable char barrier during the combustion process, which prevented heat and mass transfer and reduced the amount of flammable volatiles in the gas phase [11,41]. For PLA composites, there was no significant change compared to pristine PLA. Only a fairly low reduction in recorded values was observed in the case of samples containing 1 wt % load of FR. In this case, the pHRR value was reduced by 8.8% and THR by 10.9% in comparison to pristine PLA. However, MCC tends to be sensitive toward charring and is blind to flame inhibition, given that volatile pyrolysis products are combusted under conditions that attempt complete combustion [38]. Hence, the MCC results show an absence of any strong charring in the case of samples containing 0.5 wt % and 2 wt % load of FR. While at 0.5 wt % load, this might be the result of an insufficient amount of FR used; in case of a 2 wt % load, it could be an indication of problems with the dispersion of FR in the PLA matrix or through the gas-phase action of FR additives. Possibly, a better degree of FR dispersion in the polymer matrix (mechanical properties Table S1) could prove beneficial for improvement of fire-resistant performance. By achieving high wettability and increasing the wt % load of DOPO–HQ-functionalized graphene oxide in the PLA matrix from 2 wt % to 6 wt %, researchers were able to achieve a V-0 UL-94 rating and LOI value of 26.5% [41]. In a different scenario, a 20 wt % load of DOPO-containing flame retardant allowed the achievement of an LOI value of 35%, although this large load of FR caused a decrease in the mechanical properties of the fabricated composites [45].

### 3.3. Thermal Conductivity Testing

Thermal conductivity of composite samples was analyzed using a laser flash apparatus. Therefore, composite tablets 12.7 mm in diameter were prepared from the extruded samples. The thickness of each tablet was measured at multiple points, and an average value was calculated. In order to facilitate laser absorption onto the surface of each tablet, their surface was covered with a thin layer of graphite. The results of these measurements are presented in Table 3.

Thermal conductivity of PE samples increased with the load of FR. Even at a 1 wt % load, a significant increase (95%) in thermal conductivity was achieved. Peak value was achieved for samples with 3 wt %, load and was more than double that recorded for pristine PE. A further increase in the wt % load of FR caused a drop in thermal conductivity, although it was still close to the peak value. These results might be the effect of good dispersion of MoS_2_-based FR within the polymer matrix, which might result in further separation of few-layered MoS_2_ nanosheets, which possess good electrical and thermal conductivity [46]. In the case of the PLA composite samples modified with the addition of FRs, there was no observed change in thermal conductivity values compared to pristine PLA, regardless of wt % load. This was possibly caused by an aggregation of MoS_2_ sheets inside the polymer matrix.

## 4. Conclusions

In this work, few-layered MoS_2_ was functionalized with DOPO and introduced into PE and PLA polymer matrixes. PE samples displayed improved thermal stability through an increase in *T_10wt%_* and *T_50wt%_* values, as well as the formation of a char barrier and the disruption of heat and mass transfer that resulted in decrease of *T_max_* values. Overall, the TGA performance of PE composites under an air atmosphere was still very close to that observed for pristine PE. A significant reduction in CO emissions was observed for PE composite samples, and was nearly 99% below that of pristine PE. The combination of solid- and gas-phase action allowed for improvement in the fire-resistant properties of the PE composites. The thermal conductivity of PE composites was greatly enhanced, and was almost double that recorded for pristine polymer. In general, PLA composites displayed less impressive results in each stage, possibly due to problems with the dispersion of flame retardant within the PLA matrix.

## Figures and Tables

**Table 1:** Summary of thermogravimetric analysis (TGA) results for polyethylene (PE) and poly(lactic acid) (PLA) composites, compared to pristine polymers.

	FR Load (wt%)	*T_10wt%_* (°C)	*T_50wt%_* (°C)	*T_ma_*_x_ (°C)
PE	-	292	424	552
PE MoS_2_/Ni_2_O_3_/DOPO	1	299	422	530
	3	315	422	524
	5	322	435	526
PLA	-	333	357	499
PLA MoS_2_/Ni_2_O_3_/DOPO	0.5	330	356	495
	1	328	354	475
	2	328	353	462

**Table 2:** Microscale combustion calorimetry (MCC) combustion data of PE and PLA composites compared to pristine polymers.

	FR Load (wt%)	HRC (J g^−1^K^−1^)	pHRR (W g^−1^)	THR (kJ g^−1^)
PE	-	1222	1175	47.0
PE MoS_2_/Ni_2_O_3_/DOPO	1	1087	783	41.3
	3	978	794	40.5
	5	1004	851	41.8
PLA	-	715	573	21.8
PLA MoS_2_/Ni_2_O_3_/DOPO	0.5	719	562	20.6
	1	685	522	19.4
	2	730	564	20.6

**Table 3:** Thermal conductivity of PE and PLA composites compared to pristine polymers.

	FR Load (wt %)	Thermal Conductivity (W m^−1^K^−1^)	Increase (%)
PLA	-	0.326	-
PLA MoS_2_/Ni_2_O_3_/DOPO	0.5	0.325	0
	1	0.325	0
	2	0.326	0
PE	-	0.186	-
PE MoS_2_/Ni_2_O_3_/DOPO	1	0.363	95
	3	0.436	134
	5	0.394	112

## Data Availability

The datasets used and analyzed during the current study are available from the corresponding author on reasonable request.

## Supplementary Materials

The following are available online at https://www.mdpi.com/2079-4991/9/11/1637/s1, Figure S1: SEM images of (A) PE, (B) PE_MoS2_Ni2O3_DOPO_5%, (C) PLA, (D) PE_MoS2_Ni2O3_DOPO_2%, Table S1: Young’s modulus and tensile strength for composites, Figure S2: HRR curves of (A) PE_MoS2, (B) PLA_MoS2, (C) PE_DOPO, (D) PLA_DOPO, Table S2: MCC combustion data of PE and PLA composites in comparison to pristine polymers.

## Abbrevations

AFM	atomic force microscopy
CO	carbon oxide
DOPO	9,10-dihydro-9-oxa-10-phosphaphenanthrene-10-oxide
DOPO-MA	maleic acid functionalized DOPO
FR	flame retardant
GNS	graphene nanosheets
HRC	heat release capacity
LOI	limiting oxygen index
MCC	microscale combustion calorimetry
MoS_2_	molybdenum disulfide
Ni_2_O_3_	nickel(III) oxide
NMP	N-Methyl-2-pyrrolidone
PE	polyethylene
pHHR	peak heat release rate
PLA	poly(lactic acid)
PS	polystyrene
PVA	poly(vinyl alcohol)
TEM	transmission electron microscopy
TGA	thermogravimetric analysis
THF	tetrahydrofuran
THR	total heat release
