# Supplementary material for: DOPO-Functionalized Molybdenum Disulfide and its Impact on the Thermal Properties of Polyethylene and Poly(Lactic Acid) Composites
Source: Nanomaterials (Basel). 2019 Nov 18;9(11):1637. doi: 10.3390/nano9111637 (PMC6915400; doi:10.3390/nano9111637)
Supplement: Supplementary file 1 [file nanomaterials-09-01637-s001.pdf]

nanomaterials

MDPI

Article

DOPO-Functionalized Molybdenum Disulfide and its Impact on the Thermal Properties of Polyethylene and Poly(Lactic Acid) Composites

Karolina Wenelska [1,*], Piotr Homa [1], Stefan Popovic [2], Klaudia Maslana [1] and Ewa Mijowska [1]

[1] Nanomaterials Physicochemistry Department , Faculty of Chemical Technology and Engineering, West Pomeranian University of Technology, Piastów Ave. 42, 71-065 Szczecin, Poland; phoma@zut.edu.pl (P.H.); kmaslana@zut.edu.pl (K.M.); emijowska@zut.edu.pl (E.M.)

[2] Department of Catalysis and Chemical Reaction Engineering, National Institute of Chemistry, Hajdrihova 19, SI-1000 Ljubljana, Slovenia; popovicstefan994@gmail.com

* Correspondence: kwenelska@zut.edu.pl

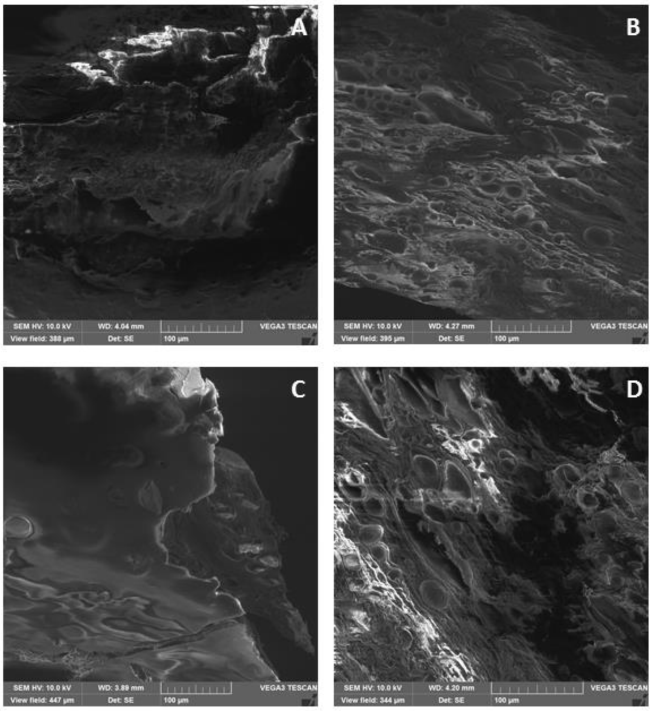

Figure S1. SEM images of (A) PE, (B) PE_MoS2_Ni2O3_DOPO_5%, (C) PLA, (D) PE_MoS2_Ni2O3_DOPO_2%.

Table S1. Young's modulus and tensile strength for composites.

Sample	Young's modulus [Mpa]	Tensile strength [Mpa]
PE	1280	34.1 ± 2.81
PE_MoS2_Ni2O3_DOPO_1%	1240	36.1 ± 2.7
PE_MoS2_Ni2O3_DOPO_3%	1150	36.4 ± 1.44
PE_MoS2_Ni2O3_DOPO_5%	868	29.0 ± 3.10
PLA	2440	59.2 ± 2.75
PLA_MoS2_Ni2O3_DOPO_0.5%	2480	59.4 ± 4.92
PLA_MoS2_Ni2O3_DOPO_1%	1820	9.32 ± 2.51
PLA_MoS2_Ni2O3_DOPO_2%	1900	11.4 ± 1.19

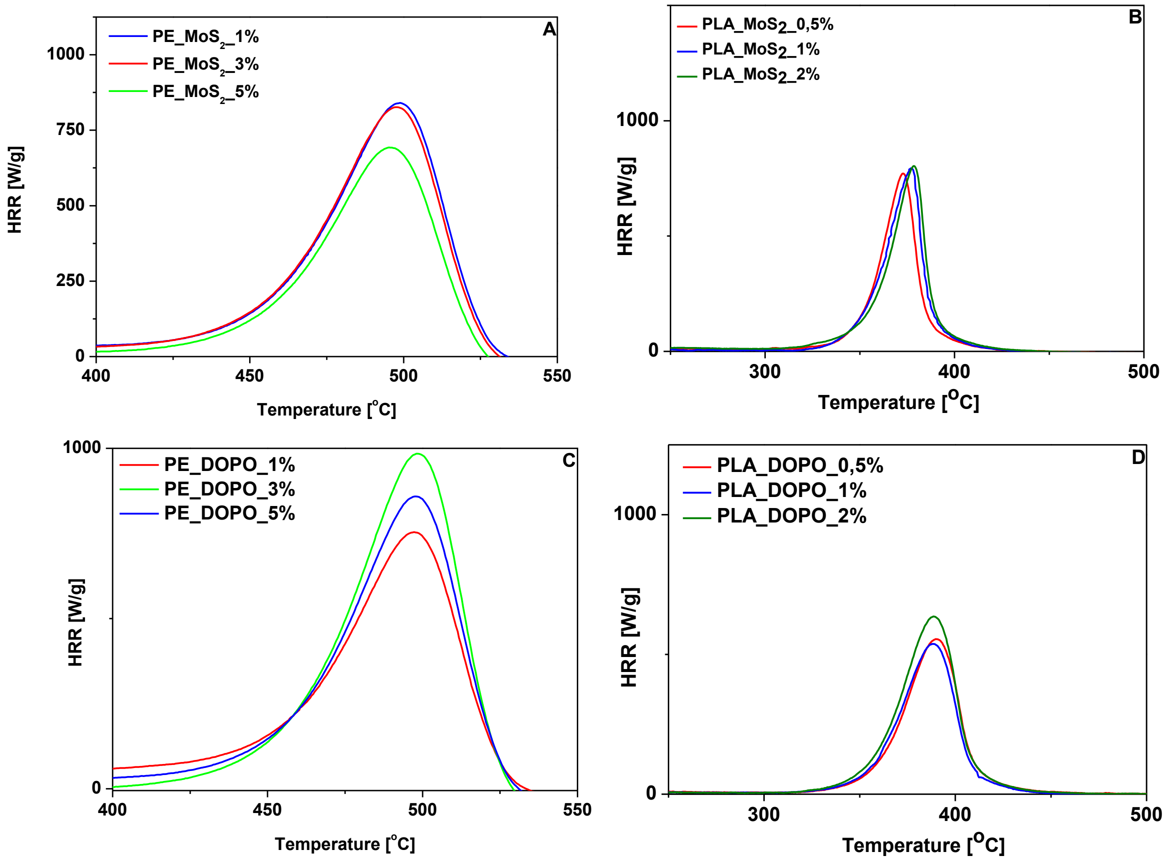

Figure S2. HRR curves of (A) PE_MoS2, (B) PLA_MoS2, (C) PE_DOPO, (D) PLA_DOPO.

Table S2. MCC combustion data of PE and PLA composites in comparison to pristine polymers.

sample	HRC [J $g^{-1}K^{-1}$]	pHRR [W g^{-1}]	THR [kJ g^{-1}]
PE	1222	1175	47.0
PE_MoS_2_1%	1009	840	45.2
PE_MoS_2_3%	1015	825	44.3
PE_MoS_2_5%	862	692	38.0
PE_DOPO_1%	934	985	46.5
PE_DOPO_3%	900	860	40.7
PE_DOPO_5%	884	752	36.4
PLA	715	573	21.8
PLA_MoS_2_0.5%	862	768	31.2
PLA_MoS_2_1%	875	796	30.8
PLA_MoS_2_2%	902	803	34.1
PLA_DOPO_0.5%	824	636	25.3
PLA_DOPO_1%	714	554	22.0
PLA_DOPO_2%	692	538	21.9

© 2019 by the authors. Submitted for possible open access publication under the terms and conditions of the Creative Commons Attribution (CC BY) license (http://creativecommons.org/licenses/by/4.0/).
